# Comparative Evaluation of the Mechanical Properties of Denture Base Resins Fabricated Using Computer-Aided Design and Manufacturing, Three-Dimensional Printing, and Conventional Heat Polymerization Techniques: An In Vitro Study

**DOI:** 10.7759/cureus.85434

**Published:** 2025-06-05

**Authors:** Pursenla Longkumer, Shashikala Jain, Navreet Bhasin, Balbir Singh, Priyanka Borse, Jasbir Kaur

**Affiliations:** 1 Department of Prosthodontics, Maharaja Ganga Singh Dental College and Research Centre, Sri Ganganagar, IND; 2 Department of Conservative Dentistry and Endodontics, Government Dental College, Amritsar, IND

**Keywords:** computer-aided design, denture bases, flexural strength, mechanical, polymethyl methacrylate, three-dimensional printing

## Abstract

Introduction: With the advent of digital dentistry, modern fabrication techniques, such as three-dimensional (3D) printing and computer-aided design/computer-aided manufacturing (CAD/CAM) milling, might offer promising alternatives to conventional heat-cured methods. This study aimed to compare the flexural and impact strengths of polymethyl methacrylate (PMMA) denture base resins fabricated using 3D printing, CAD/CAM milling, and conventional heat polymerization.

Materials and methods: An in vitro experimental design was employed to fabricate denture base resin specimens using three different techniques. Group 1 (n = 40) consisted of heat-polymerized PMMA specimens, Group 2 (n = 40) comprised CAD/CAM-milled PMMA disks, and Group 3 (n = 40) included specimens fabricated with 3D-printed PMMA resin. Standardized rectangular samples were prepared for each group and subjected to thermocycling and artificial saliva immersion to simulate oral conditions. The flexural strength was tested using a three-point bend method on a universal testing machine, while the impact strength was evaluated using an Izod-type impact testing machine. Each group consisted of 20 specimens for flexural strength testing and 20 specimens for impact strength testing. Statistical analysis was conducted using one-way analysis of variance (ANOVA) and Tukey’s post hoc test, with a significance level set at p < 0.05.

Results: Flexural strength analysis revealed that the CAD/CAM-milled specimens exhibited the highest mean strength, followed by the 3D-printed and conventional groups. Significant differences were found between the CAD/CAM and the other two groups (p < 0.05), while the 3D-printed and conventional groups showed no significant difference (p > 0.05). Impact strength testing showed that the 3D-printed specimens had the highest mean values, followed by the CAD/CAM and conventional groups. All pairwise comparisons for impact strength were statistically significant, indicating superior energy absorption in the 3D-printed specimens (p < 0.05).

Conclusion: This study demonstrated that CAD/CAM milling resulted in the highest flexural strength, making it favorable for resisting functional stress. However, the 3D-printed resins exhibited superior impact strength, suggesting enhanced resistance to sudden forces.

## Introduction

Complete tooth loss, or edentulism, poses significant challenges for many elderly individuals and profoundly affects aesthetics, phonetics, and orofacial functionality. These impairments often lead to a diminished quality of life, impacting both physical and psychological well-being [1]. Complete dentures and removable prosthetic devices crafted primarily from acrylic resins serve as cornerstone treatments for managing edentulism [2]. These dentures replace the entire dentition and associated structures of the maxilla and mandible, restoring critical oral functions such as chewing and speaking while enhancing facial aesthetics and boosting patient confidence [3]. By re-establishing oral harmony, complete dentures play a pivotal role in enabling individuals to maintain a functional and fulfilling life despite tooth loss [3].

Polymethyl methacrylate (PMMA) resin has long been the material of choice for denture base fabrication because of its favorable properties, including excellent aesthetics, low water sorption and solubility, minimal toxicity, ease of repair, and straightforward processing techniques [4]. Heat-cured PMMA, in particular, remains widely used; however, its brittleness renders acrylic dentures prone to fractures, both intraorally during function and extraorally owing to accidental impacts [5]. Studies indicate that 68% of acrylic dentures fracture within a few years of delivery, with 39% requiring repairs within three years [6]. Efforts to enhance PMMA’s durability have included reinforcement with materials such as metals, fibers, and carbon; however, challenges persist [7].

Advancements in dental technology have introduced digital fabrication methods, notably computer-aided design/computer-aided manufacturing (CAD/CAM) milling, three-dimensional (3D) printing, and revolutionizing denture production [8]. CAD/CAM milling employs subtractive manufacturing utilizing pre-polymerized PMMA disks processed under high pressure and heat to create monolithic dentures or dentures with chemically bonded teeth [9]. This approach offers superior mechanical properties, enhanced bio-hygiene owing to the hydrophobic nature of milled PMMA, and reduced risk of fracture or tooth delamination [10]. Conversely, 3D printing, an additive manufacturing technique, constructs dentures layer-by-layer from CAD-designed models, minimizing material waste and enabling cost-effective in-house production [11]. These digital methods streamline workflows, reduce fabrication steps, and enhance accessibility for clinicians [8].

The mechanical properties of denture base materials, particularly their flexural and impact strengths, are critical to their clinical success [12]. Flexural strength, encompassing compressive, tensile, and shear strengths, determines the ability of a material to withstand functional loads without fracturing. The impact strength reflects a material’s capacity to absorb energy from sudden forces, such as accidental drops, preventing breakage [12]. While numerous studies have evaluated these properties in conventional heat-cured PMMA, there is a paucity of comprehensive research comparing digitally fabricated denture bases via CAD/CAM milling and 3D printing, with their conventional counterparts [13,14]. As digital technologies are gaining prominence in dentistry, understanding the mechanical performance of these materials is essential to ensuring their suitability for clinical applications.

This study aimed to compare the flexural and impact strengths of denture base resins fabricated using 3D printing, CAD/CAM milling, and conventional heat-cure techniques. The objectives of this study were to evaluate the mechanical properties of 3D-printed PMMA, CAD/CAM-milled PMMA, and heat-cured PMMA denture bases through in vitro testing, analyze their performance under standardized conditions, and provide evidence-based insights to guide clinicians in selecting optimal materials for durable and reliable denture fabrication.

## Materials and methods

Study design

This in vitro experimental study was conducted at the Department of Prosthodontics, Maharaja Ganga Singh Dental College and Research Centre, Sri Ganganagar, Rajasthan, from May 2024 to December 2024. As this in vitro study did not involve human participants or human tissue, ethical approval was not required.

Sample size calculation

The sample size was calculated using the G*Power 3.1 software (Heinrich Heine University, Düsseldorf, Germany). The calculation was based on a significance level (α) of 0.05, power (1-β) of 0.80, and effect size derived from a pilot study. A one-way analysis of variance (ANOVA) test was used for intergroup comparisons, and the minimum required sample size was determined to be 34 per group. To accommodate for potential errors or exclusions, the final sample size was increased to 40 per group.

Group allocation

**Figure 1:**
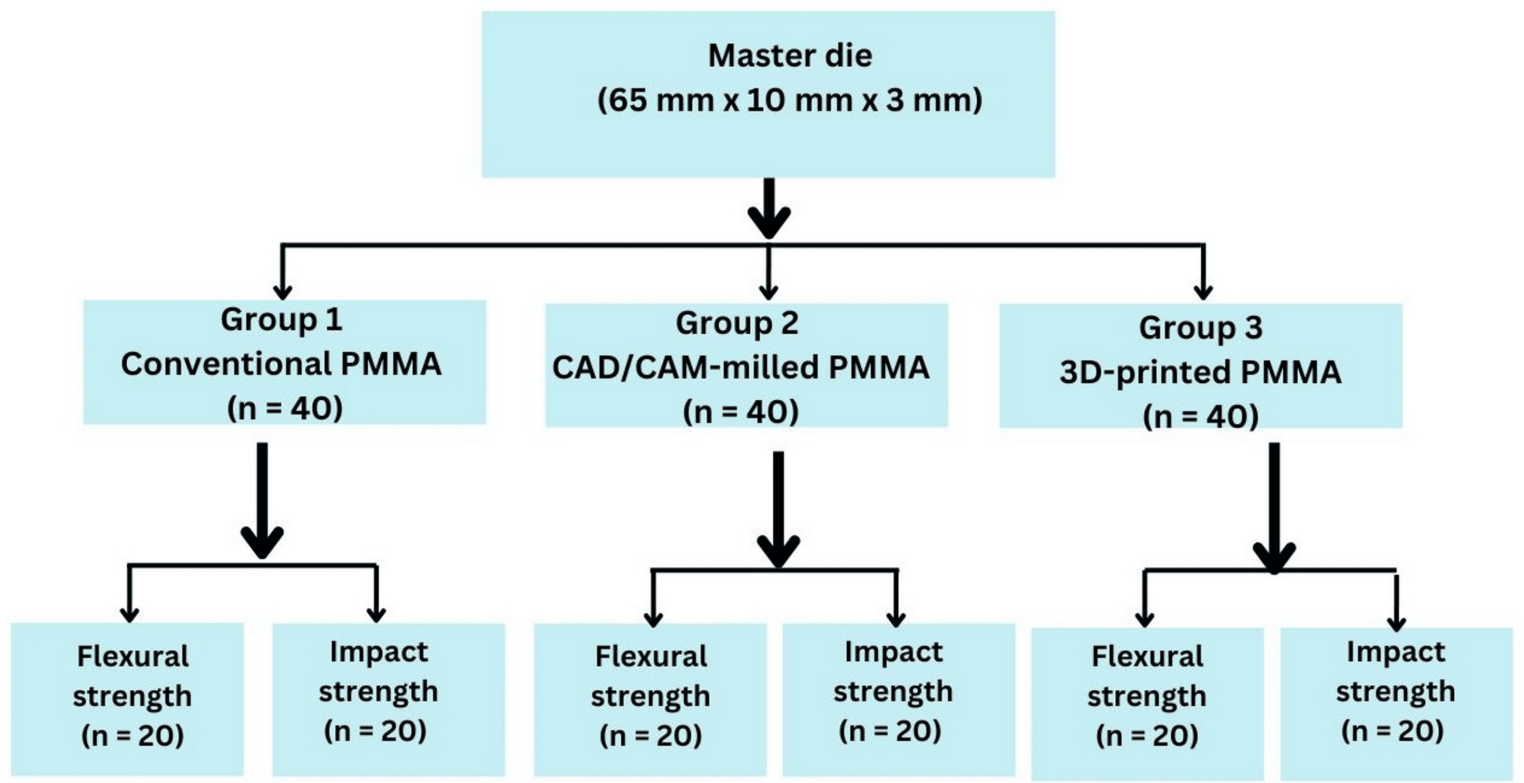
Study design and group allocation. CAD/CAM: computer-aided design/computer-aided manufacturing; 3D: three-dimensional; PMMA: polymethyl methacrylate; 3D: three-dimensional.

The specimens were divided into three groups according to the type of denture base material and the fabrication technique. Group 1 included specimens made of conventional heat-polymerized PMMA resin (Triplex Hot, Ivoclar Vivadent AG, Liechtenstein). Group 2 consisted of CAD/CAM-milled PMMA specimens fabricated from pre-polymerized disks (Ivotion Base, Ivoclar Vivadent AG, Liechtenstein). Group 3 included specimens fabricated using a 3D-printed PMMA resin (3D Accuprint Denture, D-Tech Dental Technologies, India). Each group consisted of 20 specimens for flexural strength testing and 20 specimens for impact strength testing (Figure 1).

Methodology

A standard master die with dimensions of 65 mm × 10 mm × 3 mm was digitally designed using AutoCAD 2024 (Autodesk Inc., San Rafael, CA, USA). The finalized model was exported in standard tessellation language (STL) format and milled from Plexiglass (Plazit Polygal India Pvt. Ltd., India) to serve as a template for uniform specimen fabrication across all groups.

For Group 1, the Plexiglass master die was coated with petroleum jelly and invested in a vacuum-mixed dental stone using a varsity flask. After dewaxing, two layers of die spacer were applied, and PMMA resin was mixed according to the manufacturer's guidelines. The resin was packed under 14.71 kN pressure using a hydraulic press (Force, Hi-Force Ltd., UK) and polymerized using a thermostatically controlled water bath (3 Hot, Macrodent, India) following a short curing cycle of two hours at 74°C, followed by one hour at 100°C. After polymerization, the specimens were bench-cooled, deflasked, trimmed using tungsten carbide burs, and polished with rubber acrylic burs, a pumice slurry, and a rouge. Only one surface of each specimen was polished to simulate a tissue-contact surface. The final dimensions were confirmed using a digital Vernier caliper (Mitutoyo Corporation, Japan), and the specimens were stored in distilled water for 48 hours before testing.

In Group 2, the STL file of the master die was imported into Exocad software (Align Technology, Tempe, AZ, USA), and the specimens were milled from Ivotion Base PMMA disks using a five-axis milling machine (Arum 5X-500, Doowon, South Korea) under wet conditions. The specimens were finished using tungsten carbide burs and 400-grit silicon carbide paper for 10 seconds and then polished using the same protocol as Group 1. The final dimensions were verified, and the specimens were stored in distilled water for 48 hours.

In Group 3, the STL file was transferred to a 3D printer (Phrozen, Taiwan) using digital light processing (DLP) technology. A 3D denture resin was used, and the specimens were printed with a 50 µm layer thickness. Prior to printing, the resin was agitated for three minutes, and the specimens were oriented 45° to the build platform. After printing with a UV projector (385 nm), the specimens were cleaned with isopropyl alcohol (Thermo Fisher Scientific India Pvt. Ltd., India) and post-cured for 15 minutes in a UV light-curing box (Phrozen, Taiwan). The support structures were removed, and the specimens were finished and polished according to a standard protocol. All the specimens were stored in distilled water for 48 hours prior to testing.

To evaluate the flexural strength, 20 specimens from each group, each measuring 65 × 10 × 3 mm, were subjected to a three-point loading test using a universal testing machine (Instron 3369, Instron Corporation, Norwood, MA, USA). The specimens were positioned with a span length between the supports, and a load was applied perpendicular to the center of each specimen at a deflection rate of approximately 5 mm/min, as monitored by a dial gauge. The load application continued until a deviation in the load-deflection curve was observed, indicating a specimen fracture. The flexural strength was calculated using the formula FS = 3FL/(2bd²), where FS is the flexural strength in megapascals (MPa), F is the force at break in Newtons (N), L is the span length between the supports, b is the specimen width (10 mm), and d is the specimen thickness (3 mm). This method ensured accurate measurement of the stress that each specimen could withstand before failure.

For the impact strength testing, 20 specimens from each group, each measuring 60 mm × 10 mm × 3 mm, were tested using an Izod-type impact testing machine (Tinius Olsen Model 892, Tinius Olsen Ltd., Horsham, PA, USA). Each specimen was positioned with the notch facing the pendulum hammer, and a 5.5 joules (J) pendulum hammer was used to deliver a sudden force at the center of the specimen from the notched side. After accounting for an attrition value of 0.01 J, the net energy absorbed (Ec) of each specimen was recorded. The impact strength was calculated using the formula I = Ec/(WT), where I is the impact strength in kJ/m², Ec is the net energy absorbed in joules, W is the specimen width in meters, and T is the specimen thickness in meters. This approach allowed for the precise determination of the energy absorption capacity of denture base materials under sudden impact.

Statistical analysis

All collected data were statistically analyzed using the Statistical Package for the Social Sciences (SPSS) software (version 26.0; IBM Corp., Armonk, NY, USA). Descriptive statistics, including the mean and standard deviation, were calculated. The Shapiro-Wilk test was used to assess the normality of data distribution, and Levene’s test was applied to evaluate the homogeneity of variance. As the data were found to be normally distributed, one-way ANOVA followed by Tukey's honestly significant difference (HSD) post hoc test was used for intergroup comparisons. Statistical significance was set at p < 0.05.

## Results

ANOVA test results revealed significant differences in the mean flexural strength (MPa) among the three study groups (F = 17.63, p = 0.001). The CAD/CAM group exhibited the highest mean strength (105.5 ± 7.61 MPa), followed by the 3D-printed group (95.89 ± 2.55 MPa) and the conventional PMMA group (94.2 ± 7.09 MPa). These findings suggested that CAD/CAM fabrication provided significantly higher flexural strength, while 3D printing offered greater precision than conventional techniques (Table 1).

**Table 1:** Comparison of mean flexural strength (MPa) between study groups using the one-way analysis of variance (ANOVA) test. *p-value < 0.05: significant. CAD/CAM: computer-aided design/computer-aided manufacturing; 3D: three-dimensional; PMMA: polymethyl methacrylate; CI: confidence interval; MPa: megapascals. Data are presented in the form of mean and standard deviation (SD).

Groups	n (%)	Mean ± SD	95% CI at mean		F stats	p-value
			Lower limit	Upper limit		
Conventional PMMA	20 (33.33%)	94.2 ± 7.09	90.98	97.61	17.63	0.001*
CAD/CAM	20 (33.33%)	105.5 ± 7.61	101.49	108.62		
3D-printed	20 (33.33%)	95.89 ± 2.55	94.71	97.09		

Post hoc Tukey’s test further revealed significant pairwise differences in flexural strength (MPa) between the groups. The CAD/CAM group demonstrated significantly higher strength than both the conventional PMMA (mean difference = 11.3, p = 0.001) and 3D-printed (mean difference = 9.61, p = 0.001) groups. In contrast, no significant difference was found between the conventional PMMA and 3D-printed groups (mean difference = 1.69, p = 0.693). These results confirmed that CAD/CAM restorations showed superior flexural strength, whereas 3D-printed and conventionally fabricated PMMA materials performed similarly in terms of mechanical resistance under flexural stress (Table 2).

**Table 2:** Pairwise comparison of study groups for flexural strength (MPa) using post hoc Tukey’s test. *p-value < 0.05: significant. CAD/CAM: computer-aided design/computer-aided manufacturing; 3D: three-dimensional; PMMA: polymethyl methacrylate; MPa: megapascals; CI: confidence interval.

Pairwise comparison		Mean difference	Standard error	95% CI at mean		p-value
				Lower	Upper	
Conventional PMMA	CAD/CAM	11.3	1.95	6.59	16.05	0.001*
Conventional PMMA	3D-printed	1.69	1.95	-3.01	6.39	0.693
CAD/CAM	3D-printed	9.61	1.95	-14.32	-4.9	0.001*

The ANOVA test revealed highly significant differences in the mean impact strength (kJ/m^2^) among the three groups (F = 228.4, p = 0.001). The 3D-printed group demonstrated the highest impact strength (3.43 ± 0.12 kJ/m^2^), followed by CAD/CAM (2.59 ± 0.26 kJ/m^2^) and conventional (2.06 ± 0.2 kJ/m^2^). Notably, the 3D-printed group showed superior strength and lowest variability, suggesting a more consistent performance. These findings indicated that additive manufacturing techniques enhanced the impact resistance compared to subtractive CAD/CAM and conventional PMMA fabrication methods, potentially due to differences in material properties or structural integrity from the manufacturing process (Table 3).

**Table 3:** Comparison of mean impact strength (kJ/m2) between study groups using the one-way analysis of variance (ANOVA) test. *p-value < 0.05: significant. CAD/CAM: computer-aided design/computer-aided manufacturing; 3D: three-dimensional; PMMA: polymethyl methacrylate; CI: confidence interval. Data are presented in the form of mean and standard deviation (SD).

Groups	n (%)	Mean ± SD	95% CI at mean		F stats	p-value
			Lower limit	Upper limit		
Conventional PMMA	20 (33.33%)	2.06 ± 0.20	1.96	2.15	228.4	0.001*
CAD/CAM	20 (33.33%)	2.59 ± 0.26	2.46	2.71		
3D-printed	20 (33.33%)	3.43 ± 0.12	3.37	3.48		

Post hoc Tukey’s test for impact strength (kJ/m^2^) revealed statistically significant differences (p = 0.001) between all pairwise comparisons. The 3D-printed group showed the highest impact resistance compared to the CAD/CAM group. The CAD/CAM group also outperformed the conventional PMMA fabrication. These findings confirmed that 3D-printed materials possessed superior impact strength, followed by CAD/CAM, whereas conventional methods exhibited the lowest resistance to impact forces (Table 4).

**Table 4:** Pairwise comparison of study groups using post hoc Tukey’s test. *p-value < 0.05: significant. CAD/CAM: computer-aided design/computer-aided manufacturing; 3D: three-dimensional; PMMA: polymethyl methacrylate; CI: confidence interval.

Pairwise comparison		Mean difference	Standard error	95% CI at mean		p-value
				Lower	Upper	
Conventional PMMA	CAD/CAM	0.53	0.064	0.37	0.68	0.001*
Conventional PMMA	3D-printed	1.37	0.064	1.21	1.52	0.001*
CAD/CAM	3D-printed	0.84	0.064	0.68	0.99	0.001*

## Discussion

PMMA resin has long been the cornerstone material for denture base fabrication because of its favorable aesthetics, ease of processing, reparability, and cost-effectiveness [4]. However, their susceptibility to fracture under impact and flexural forces, attributed to dimensional distortion and processing errors, limits their longevity [5]. The advent of digital fabrication techniques, such as CAD/CAM milling and 3D printing, has addressed these shortcomings by enhancing the mechanical properties of PMMA denture bases [8]. The findings of this study demonstrated that CAD/CAM-milled PMMA exhibited the highest flexural strength, followed by 3D-printed PMMA, and conventional heat-cured PMMA showed the lowest strength. These results are consistent with previous research and highlight the mechanical superiority of digitally fabricated denture bases, offering valuable insights into their clinical applicability [12-14].

The superior flexural strength of the CAD/CAM-milled PMMA, with values consistent with those of Prpić et al. [15] and Al-Dwairi et al. [16], can be attributed to its industrial pre-polymerization process. This process, conducted under high temperature and pressure, produces a dense polymer structure with minimal porosity, reduced polymerization shrinkage, and low residual monomer content [10]. The monolithic design of CAD/CAM-milled dentures further reduces the risk of tooth delamination and improves structural integrity, making them highly durable under masticatory loads [10]. In contrast, conventional heat-cured PMMA exhibits the lowest flexural strength, likely because of the porosity formed during the curing cycle [15]. The boiling of residual methyl methacrylate monomers creates internal voids, which act as stress concentrators and facilitate crack propagation, as reported in previous studies [15,16].

3D-printed PMMA, while demonstrating lower flexural strength than CAD/CAM-milled PMMA, outperformed the conventional heat-cured PMMA. The lower flexural strength of 3D-printed PMMA may be attributed to the incremental layering inherent in additive manufacturing, which can initiate crack propagation and compromise the structural integrity [13]. However, the impact strength of 3D-printed PMMA was notably higher owing to the optimized printing parameters, including a 45° orientation and a 50% rectilinear infill pattern [13]. These parameters, supported by Al-Dwairi et al. [16], enhance the impact resistance by improving the stress distribution and structural continuity. The rectilinear infill pattern forms continuous layers that increase energy absorption, making 3D-printed dentures more resilient to sudden impacts such as accidental drops [17]. This finding underscores the potential of 3D printing to produce denture bases with balanced mechanical properties, particularly in scenarios in which impact resistance is critical.

The impact strength of CAD/CAM-milled PMMA also surpassed that of conventional heat-cured PMMA, likely owing to its high degree of polymerization [18]. The pre-polymerized PMMA blocks used in CAD/CAM milling are processed using sophisticated equipment, resulting in a condensed resin mass with minimal porosity and residual monomers. This is in contrast to conventional techniques, where manual mixing and inconsistent pressure during polymerization lead to defects such as internal voids, reducing impact resistance. The findings of this study align with those of Steinmassl et al. [19], who noted variations in physical properties among CAD/CAM PMMA brands owing to differences in packing density and porosity.

The differences in the mechanical performance among the tested groups highlighted the influence of the fabrication techniques on PMMA’s internal structure of PMMA. CAD/CAM milling produces a highly uniform and dense material that is ideal for withstanding flexural stresses, whereas 3D printing offers flexibility in design and enhanced impact resistance through customizable infill patterns [15]. Despite meeting international standards, conventional heat-cured PMMA is limited by its susceptibility to processing defects [15]. All tested specimens exceeded the International Organization for Standardization (ISO) 20795-1 minimum flexural strength requirement of 65 MPa and the American Dental Association (ADA) Specification No. 12 impact strength threshold, confirming their suitability for clinical use. However, the superior mechanical properties of CAD/CAM-milled and 3D-printed PMMA suggest that digital fabrication techniques may offer greater long-term reliability [8].

Clinical implications

The findings of this study have significant implications for clinical practice. The superior flexural strength of CAD/CAM-milled PMMA makes it an ideal choice for patients with high masticatory demands or those prone to denture fractures due to heavy occlusal forces. Their bio-hygienic properties and durability enhance patient outcomes by reducing maintenance needs and improving prosthesis longevity. 3D-printed PMMA, with its enhanced impact strength, is well suited for patients at risk of accidental denture drops, such as elderly individuals with reduced dexterity. The cost-effectiveness and accessibility of 3D printing also enable in-house fabrication, potentially reducing turnaround time and laboratory costs. Both digital techniques streamline workflows, offering clinicians greater precision and customization than conventional methods. By selecting an appropriate fabrication technique based on patient-specific needs, clinicians can optimize functional and aesthetic outcomes, ultimately improving patient satisfaction and quality of life.

Limitations

Despite these promising results, this study had several limitations. The use of a single CAD/CAM PMMA brand might limit the generalizability of the findings because variations in material composition and manufacturing standards across brands could affect mechanical properties, as noted by Steinmassl et al. [19]. Additionally, the in vitro nature of this study does not fully replicate the complex intraoral environment, where factors such as saliva, temperature fluctuations, and cyclic loading may influence material performance. The 3D-printed PMMA specimens were tested with specific printing parameters (45° orientation and 50% rectilinear infill), and different settings yielded varying results. Furthermore, long-term clinical studies are required to assess the durability and performance of digitally fabricated dentures under real-world conditions. Future studies should explore the impact of different PMMA brands, printing parameters, and intraoral factors to provide a more comprehensive understanding of the clinical efficacy of these materials.

## Conclusions

This study compares the flexural and impact strengths of denture base resins fabricated using conventional heat-cured, CAD/CAM-milled, and 3D-printed PMMA. The CAD/CAM-milled PMMA demonstrated superior flexural strength owing to its dense polymer structure achieved through controlled pre-polymerization. The 3D-printed PMMA exhibited the highest impact strength, which was enhanced by the optimized printing parameters that improved energy absorption. Conventional heat-cured PMMA showed the lowest performance in both properties, likely owing to processing-induced porosity. All materials met the international standards for clinical use, confirming their suitability for denture fabrication. The findings highlight the mechanical advantages of digital fabrication techniques, with CAD/CAM milling offering exceptional durability and 3D printing providing enhanced impact resistance.
